# A Case of Diffuse Brucellar Spondylodiscitis

**DOI:** 10.7759/cureus.17874

**Published:** 2021-09-10

**Authors:** Suhas P Dasari, Mit Patel, Vishmayaa Saravanan, Ross Rybakowicz, Pinky Jha

**Affiliations:** 1 Internal Medicine, Medical College of Wisconsin, Wauwatosa, USA

**Keywords:** brucella, spondylodiscitis, piriformis abscess, diffuse brucellosis, discitis

## Abstract

Diffuse brucellar spondylodiscitis is the most severe subtype of osteoarticular brucellosis and is defined as a brucellar infection involving an entire vertebral body, typically a lumbar vertebra, with spread to the adjacent disc space, vertebra, and even extravertebral spaces, including epidural, paraspinal, or intramuscular locations. Although it is a relatively rare diagnosis in the US healthcare system, it should be considered in all patients with severe back pain, radicular symptoms, and a history of extensive exposure to an endemic area. Any delays in treatment can be associated with an increased risk of permanent neurological deficits or death. Here, we present a case of diffuse brucellar spondylodiscitis in a patient who presented to our facility with a history of extensive exposure to an endemic area. While an MRI can reveal pathognomonic findings in brucellar spondylodiscitis, for our case, it was nonspecific. The MRI provided early evidence of an infectious etiology which prompted immediate broad-spectrum antimicrobial coverage until causal organisms were identified and culture sensitivities directed targeted antibiotic therapy. The patient was able to recover over the course of four months without surgical intervention. At her final clinical follow-up, she had no neurological deficits and had complete resolution of her radicular symptoms.

## Introduction

Brucellosis is an endemic microbial infection caused by *Brucella*, a gram-negative coccobacillus. Humans typically develop brucellosis through the ingestion of contaminated dairy products or contact with infected livestock. Patients initially present with nonspecific symptoms and laboratory findings such as anemia, leukopenia, and thrombocytopenia. For this reason, the diagnosis may be complicated and heavily reliant on patient history. The concomitant presence of osteoarticular complications may help narrow the differential diagnoses.

Brucellar discitis refers to a specific inflammation of the intervertebral disc, and its extension into the vertebral body is termed spondylodiscitis [1]. This spinal involvement with lower back pain and lumbar involvement often leads to the false diagnosis of sciatica or herniated discs due to similar presentation [2]. Fortunately, modern imaging has made this misdiagnosis less likely to occur and can give early suspicion to an infectious etiology. In cases of focal complications where there is clinical suspicion, MRI can be an essential diagnostic tool. An MRI helps in the diagnosis of brucellar spondylodiscitis and can show characteristic anterior superior end erosion, which is a pathognomonic finding termed the Pedro Pons’ sign [1-5]. In cases where an MRI does not show this pathognomonic finding, it can still be of critical importance by revealing an infectious etiology, which will expedite the initiation of broad-spectrum antimicrobials.

Brucellosis is most commonly seen in Asian countries such as India, China, and Thailand [6]. However, frequent travel from endemic areas has increased the geographic range of brucellosis. The incubation period of *Brucella* ranges from two weeks to six months, thus obtaining an extensive travel history from potential patients is crucial in diagnosis [6]. Nonspecific back pain is the main presenting symptom of spondylodiscitis, but its lack of specificity often leads to delays in diagnosis and treatment, which can prove to be permanently disabling or even fatal as the management of brucellar spondylodiscitis is time-sensitive [1]. While rare in the US healthcare system, it is necessary to consider brucellar spondylodiscitis in patients with severe and persistent cervical, lumbar, and/or sacral pain and a history that would put them at risk for exposure [1]. We present this case in an effort to increase the awareness and understanding of diagnosing and managing brucellar spondylodiscitis within the US health care system.

## Case presentation

A 55-year-old female patient from Ecuador presented with three months of left radicular pain, bilateral hip pain, and severe lower back pain, which waxed and waned intermittently. She presented to our facility without prior imaging. Upon presentation, the patient was afebrile with stable vitals and complaints of bilateral lower extremity numbness, tingling, and shooting pain without a particular dermatomal distribution. She was limited by pain upon ambulation and reported intermittent fevers without bladder/bowel incontinence or upper extremity symptoms. Overall, she was grossly neurologically intact. She was hyponatremic (123 mmol/L) with an elevated C-reactive protein (5.4 mg/dL) and had benign remaining labs including creatinine (0.63 mg/dL) and white blood cell count (5.9 × 10^3^ cells/uL). Our initial differential diagnosis included a variety of noninfectious pathologies including spondylosis, spinal stenosis, compression fractures, and facet joint arthropathy, as well as more common infectious etiologies including typical gram-positive (*Staphylococcus* and *Streptococcus* species) and gram-negative species (*Escherichia coli*, *Klebsiella*, and *Salmonella*) [7]. Although atypical infectious etiologies were low on the differential diagnosis, they were considered due to the patient’s history of extensive exposure to an endemic area.

**Figure 1:**
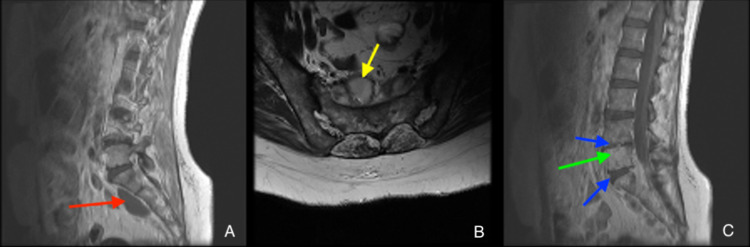
MRI upon admission. Contrast-enhanced sagittal T1 (A), axial T2 (B), and sagittal STIR (C) showing osteodiscitis centered around L5-S1 (green arrow), loss of disc height at L4-L5 and L5-S1 (blue arrows), rim-enhancing abscess anterior to the sacrum (red arrow), and rim-enhancing lesion in the right piriformis (yellow arrow). MRI: magnetic resonance imaging; STIR: short tau inversion recovery

The initial MRI upon admission showed extensive hyperintensities of the vertebral body on T2 scans, indicative of spondylodiscitis centered around L5-S1 with bilateral epidural, paraspinal, and intramuscular abscesses (Figure 1). The largest abscess was centered in the left piriformis. Blood cultures were drawn, and the patient underwent a CT-guided biopsy and aspiration of the left piriformis muscle abscess. Although these findings were nonspecific for a single pathogen, when combined with her history of residence outside of the United States, there was high suspicion for an atypical infection. As no specific causal organism was identified, she was started on intravenous (IV) vancomycin and ceftriaxone but continued to worsen with excruciating pain refractory to hydromorphone. Vancomycin was chosen for gram-positive coverage as a large proportion of pyogenic spondylodiscitis cases are caused by *S. aureus*, and, therefore, in light of no clear causal organism, nonspecific broad coverage was pursued [7].

**Figure 2:**
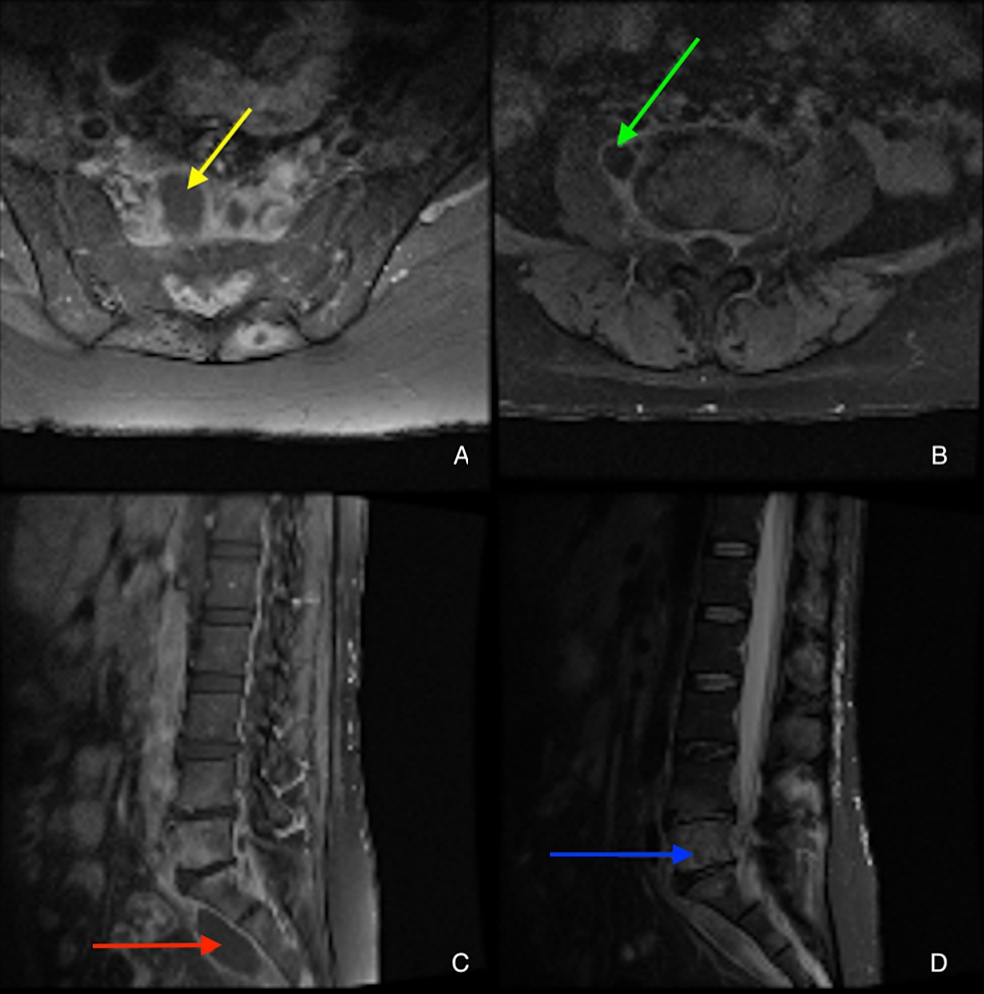
MRI after one week of treatment (10 days after admission). Contrast-enhanced axial T1 image showing presacral abscess (A, yellow arrow). Contrast-enhanced axial T1 image showing right psoas abscess (B, green arrow). Contrast-enhanced sagittal T1 image showing presacral abscess (C, red arrow). Sagittal STIR marrow showing hyperfluorescence in L5 vertebral body spreading to L4 and S1, indicative of an infectious process (D, blue arrow). MRI: magnetic resonance imaging; STIR: short tau inversion recovery

**Figure 3:**
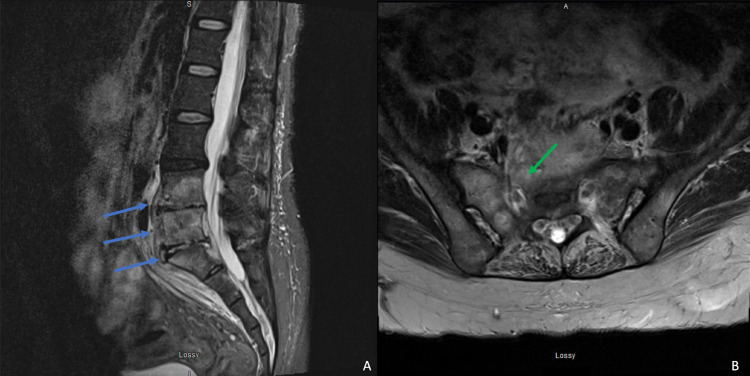
MRI at four months. Sagittal STIR (A) and axial T2 (B) images revealing stabilization of lesions in L4-S1. MRI: magnetic resonance imaging; STIR: short tau inversion recovery

On day three after admission, one of the two initial blood cultures began to grow gram-negative rods, and, therefore, ceftriaxone was switched to piperacillin/tazobactam for better *Pseudomonas* and anaerobic coverage. Blood cultures grew *Brucella melitensis* on day six, and, upon further questioning, the patient admitted to a history of consuming unpasteurized cheese and working with sheep and other livestock. Upon identification of the causal organism, vancomycin was discontinued. On day eight, biopsy culture of the left gluteal abscess grew *Brucella* species as well. A repeated MRI showed a stable osteodiscitis, a stable epidural abscess, and new/growing paraspinal and intramuscular abscesses (Figure 2). Over her hospital course, she clinically improved while on piperacillin/tazobactam. She was then transitioned to doxycycline, rifampin, and gentamicin based on the in-vitro sensitivity profile determined from biopsied samples. She also developed cellulitis which was treated with cephalexin for seven days. The patient’s creatinine continued to rise and her acute kidney injury (AKI) was attributed to the nephrotoxicity of gentamicin which was subsequently replaced with ciprofloxacin. Subsequently, the patient’s kidney function improved. The patient was discharged to a rehabilitation facility on doxycycline and rifampin for three more months and was doing well with improved mobility, reduced numbness, and reduced tingling; however, she continues to complain of left piriformis pain. Upon her most recent clinical follow-up four months after the initial presentation, she is ambulating on her own without assistive devices, has greatly improved pain in her hips and back, and has no complaints of numbness, radiating pain, or tingling. Her MRI at this time is shown in Figure 3 and reveals stabilization of her vertebral and presacral lesions.

## Discussion

Here, we present the case of a patient from South America who presented in the United States with complaints of chronic back pain and radicular symptoms. She was ultimately diagnosed with diffuse brucellar spondylodiscitis. The patient only improved after she had been transitioned to antimicrobials with broad-spectrum gram-negative coverage upon identification of gram-negative rods. She continued to improve with targeted therapy once antibiotic susceptibilities were determined. Due to the time-sensitive nature of spondylodiscitis, delays in her care could have led to a permanent neurological deficit. Fortunately, she improved after a long course with multiple antibiotics but there was still significant morbidity associated due to her long hospitalization and rehabilitation. For these cases, early infectious disease evaluation is critical for quick diagnosis and management, especially in those with osteoarticular manifestations, to prevent the adverse effects caused by potentially fatal delays in treatment [1]. It is possible that earlier treatment could have led to reduced morbidity experienced by the patient. For this case, imaging findings and biopsy results allowed for the implementation of an adequate antibiotic management plan for this patient’s complicated and rare presentation.

Pathophysiology

*Brucella*, a small, intracellular, gram-negative coccobacillus, is responsible for causing brucellosis in humans, with *B. melitensis *being the most virulent and invasive member of the genus [8,9]. Although the disease is frequently caused by direct human contact with infected animals, transmission can also occur through the ingestion of unpasteurized dairy products [10]. Through hematogenous spread, chronic brucellosis can reach the lymph nodes, spleen, liver, bone marrow, mammary glands, and sex organs. Specifically, complications arising from bone marrow infection may cause spinal brucellosis, the most common site of musculoskeletal involvement. This can present as either spondylitis, spondylodiscitis, and/or discitis, usually involving the lumbar area [11,12].

In the case of spondylodiscitis, the vertebrae and disc are simultaneously inflamed. This is capable of producing severe lower back, radicular, and hip pain, as well as neurologic symptoms such as various forms of paresthesia affecting the lower extremities. Spondylodiscitis may be seen as having single-focal, contiguous multifocal, or noncontiguous multifocal involvement. The focal form is confined to the anterior portion of an endplate which typically occurs at the anterior superior endplate of a lumbar vertebra, an area known for rich blood supply allowing the hematogenous spread of this bacteria. The diffuse form may involve the entire vertebral body and extend to the adjacent disc, vertebrae, and epidural space [13,14]. This is considered the most severe form of osteoarticular involvement of brucellosis because of the high incidence of skeletal and neurological sequelae despite multiple treatment regimens [15]. Our patient likely had the diffuse form of brucellar spondylodiscitis due to the presence of paraspinal, intramuscular, and epidural abscesses, as well as the nearly complete involvement of the L5 vertebral body with spread into the adjacent disc space and vertebra (Figure 2, bottom right).

Fortunately, despite having such a severe presentation, she was able to respond to treatment options that had gram-negative coverage and was able to recover with no permanent skeletal or neurological injuries. She required multiple treatment regimens, but this was mainly due to her AKI rather than a lack of antimicrobial response.

Diagnosis

The presentation of brucellosis is variable and nonspecific [16]. It is notable, however, that backache has such a high prevalence in these patients that brucellosis, while rare, may be considered in the differential diagnosis for any patient with severe sciatic or back pain and extensive exposure to an endemic area [1]. The management of spondylodiscitis is heavily dependent on the diagnostic power that MRI provides, and, according to Ganji et al., MRI is the best method to “localize the cause of spondylodiscitis, epidural abscess, or compression on the spine and spinal nerves related to brucellosis” [1]. Epidural abscesses are a rare complication of spinal brucellosis, but it is critical to not overlook their presence as delayed treatment can lead to rapidly progressing severe outcomes including permanent neurological deficits and potentially even death [1]. MRI is also useful in differentiating brucellar spondylodiscitis from tuberculous spondylitis, pyogenic spondylitis, postoperative changes, and other common spinal pathologies [17]. The pathognomonic imaging finding for brucellar spondylodiscitis is a destructive lesion in the anterosuperior vertebral corner with prominent osteosclerosis [1], which is termed the Pedro Pons’ sign. Taken together, MRI can help guide early antimicrobial therapy until the causal organism can be identified on culture. While MRI has been reported to be pathognomonic for the diagnosis of brucellar spondylodiscitis, in this case, there was nonspecific diffuse sclerosis of the vertebra and diagnosis was dependent on culture findings.

Treatment

The prognosis of spondylodiscitis had been poor until the advent of antimicrobial agents. Despite this, current studies report high morbidity and mortality associated with spondylodiscitis. A systematic literature review by Sobottke et al. in 2008 noted that patients with spondylodiscitis spent a mean time of up to 57 days in the hospital with mortality as high as 17% [18]. Currently, the treatment for brucellar spondylodiscitis is usually up to three months to attempt to prevent relapses [1]. Commonly used antimicrobial agents include doxycycline, streptomycin, gentamicin, ciprofloxacin, trimethoprim/sulfamethoxazole, and rifampin [1]. The recommended treatment plan includes a combination of two or three antibiotic agents, with the most popular being streptomycin plus doxycycline. Our patient improved initially on piperacillin/tazobactam, but the majority of her treatment was based on a regimen using doxycycline, gentamicin, and rifampin. As her AKI worsened, she was transitioned to ciprofloxacin, which allowed her to continue to improve. Her overall therapy lasted roughly four months, and she still requires extensive work with therapy and rehabilitation in Ecuador. For these cases, surgery is only indicated for patients with persistent spondylodiscitis that has proven refractory to multiple antibiotic regimens, those with epidural or spinal abscesses, or those presenting with progressively worsening neurological deficits [1,19]. While our patient had indications for surgery due to her epidural abscesses, her clinical presentation and lack of focal neurological deficits or progression of symptoms warranted us to pursue a nonoperative treatment plan that was successful despite her delayed diagnosis of diffuse brucellar spondylodiscitis.

## Conclusions

While a rare diagnosis in the US healthcare system, brucellar spondylodiscitis should not be missed by physicians as it can rapidly lead to permanently debilitating outcomes. A history of extensive exposure to an endemic area should provide a suspicion of atypical pathogens causing spondylodiscitis, and MRI can be used to differentiate brucellosis from other infectious etiologies unusual to the United States such as Pott’s disease. These along with the clinical presentation can help guide the antimicrobial treatment. While MRI plays a critical role in the diagnosis of diffuse brucellosis in the literature, in our case, MRI findings did not confirm the diagnosis. Instead, the imaging findings confirmed the clinical suspicion of an infectious etiology, allowing us to initiate broad-spectrum antibiotics while awaiting confirmatory biopsy findings. For this case, a definitive diagnosis was determined with biopsy culture results. Through this report, we hope to increase the awareness of atypical spondylodiscitis within our healthcare system, describe a case where multidrug regimens with strong gram-negative coverage adequately treated diffuse brucellar spondylodiscitis without surgical intervention, and emphasize the importance of patient history when assessing chronic low back pain with radicular symptoms.
